# Multifocal Metachronous Giant Cell Tumor: Case Report and Review of the Literature

**DOI:** 10.1155/2014/678035

**Published:** 2014-01-05

**Authors:** B. Ghostine, A. Sebaaly, I. Ghanem

**Affiliations:** Department of Orthopedic Surgery, Hotel Dieu de France Hospital, Alfred Naccache Street, Achrafieh, P.O. Box 166830, Beirut, Lebanon

## Abstract

*Introduction*. Giant cell tumors (GCTs) of bone are known for their local aggressiveness and high recurrence rate. There are rare cases of multicentric GCT and most are synchronous. We herein review metachronous multicentric GCT reported in the literature. *Material and Methods*. A MEDLINE, Cochrane, and Google Scholar search was done to collect all cases of multicentric metachronous GCT specifying the clinical, radiological, and histological characteristics of each location and its treatment. *Results*. A total of 37 multifocal giant cell tumors were found in the literature. 68% of cases of multicentric giant cell tumors occur in less than 4 years following treatment of the first lesion. Thirty-seven cases of multifocal metachronous GCT were identified in the literature until 2012. Patients with multicentric GCT tend to be younger averaging 23. There is a slight female predominance in metachronous GCT. The most common site of the primary GCT is around the knee followed by wrist and hand and feet. Recurrence rate of multicentric GCT is 28.5%. *Conclusion*. Multicentric giant cell tumor is rare. The correct diagnosis relies on correlation of clinical and radiographic findings with confirmation of the diagnosis by histopathologic examination.

## 1. Introduction

Giant cell tumors (GCTs) of bone are known for their local aggressiveness and high recurrence rate. Patients with GCT present with nonspecific symptoms including pain, overlying soft-tissue swelling, and decreased range of motion at the adjacent joint [1].

They rarely metastasize to distant structures such as the lung, although these metastases generally have the same benign histologic appearance as the index tumor [2]. Even rarer are cases of multicentric giant cell tumor. Most multicentric giant cell tumors are synchronous, that is, occurring within a poorly defined time of the initial tumor [3].

In this paper, we present the case of a metachronous giant cell tumor as well as a review of the literature of metachronous multicentric giant cell tumor.

## 2. Case Report

**Figure 1:**
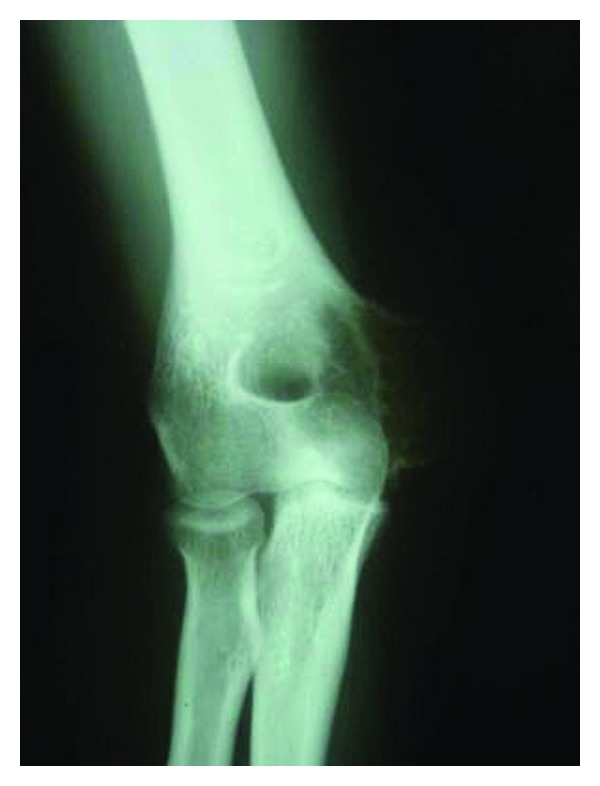
AP view of the elbow with radiolucent lesion of the medial condyle.

An 18-year-old female presented to our institution with inflammatory right elbow pain and elbow stiffness. X-rays showed a lucent image on the medial aspect of the right distal humeral metaphysis with a radiologically intact cortex (Figure 1). Histological diagnosis of GCT was made on open biopsy. Extensive curettage was undertaken, phenol was applied on the walls of the remaining cavity as well as high-speed burring, and the cavity was filled with methyl methacrylate, with a satisfying result.

Four years later, X-rays showed involvement of the lateral condyle and MRI articular involvement. The diagnosis of recurrent GCT was confirmed on biopsy. A total marginal excision of the elbow joint was undertaken along with prosthetic elbow arthroplasty. Three years following the surgery, the patient was free of tumor and pain but had an unstable elbow due to prosthetic dislocation, but she said that she was satisfied with the result and refused revision surgery.

**Figure 2:**
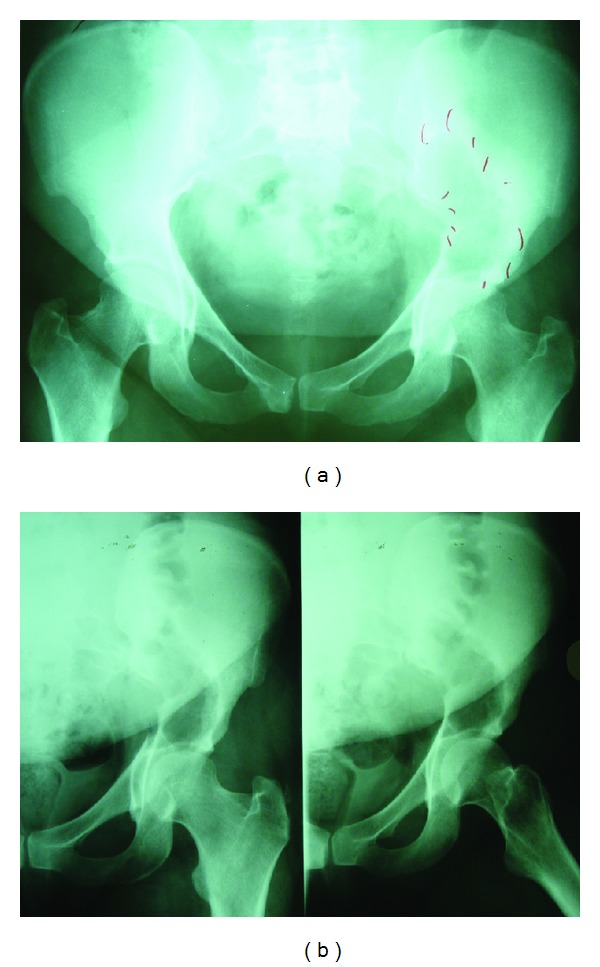
AP view of the pelvis showing a lucent lesion of the supra-acetabular area of the left iliac bone.

She was then lost to followup and came back only 7 years later, at the age of 32, after a fall from the stairs with pain around the left hip persisting for several weeks despite a regular use of anti-inflammatories and pain killers. Pelvic radiographs showed a lucent lesion of the left iliac bone (Figures 2(a) and 2(b)). MRI showed active lytic process occupying the left iliac wing without cortical or joint involvement. PET-CT scan showed a high uptake of left iliac wing (6.8 SUV uptake) with no other involvement sites. Parathyroid hormone levels were normal. Biopsy confirmed GCT. She was operated on with curettage, application of phenol and alcohol, and methyl methacrylate. Followup after two years was clinically and radiologically unremarkable and a chest CT scan one year later showed no lung metastases.

## 3. Material and Methods

A MEDLINE, Cochrane, and Google Scholar search was done using the following keywords: metachronous giant cell tumor. Inclusion criteria were (1) case report of metachronous multifocal GCT; (2) histologically confirmed GCT. The first distinction between synchronous and metachronous GCT was provided by Park et al. who defined a metachronous GCT as a GCT with 2 or more locations separated by more than 6 months in presentation [4]. Clinical characteristics of each case, involvement sites, radiographic findings, treatment modalities, and recurrences were recorded (Table 1).

## 4. Results (Table 1)

A total of 37 cases of multifocal metachronous GCT were identified until 2012. There were 15 (40%) male and 22 (60%) female patients. Average age at diagnosis of the first GCT was 23 years.

The site of first involvement was in the upper extremity in 16 cases: 4 in the distal ulna, 4 in the distal radius, 5 in the humerus (4 proximal and 1 distal), and 3 in the hand, and in the lower extremity in 19 patients: 7 in the distal femur, 7 in the proximal tibia, 3 in the proximal fibula, and 2 in the foot. One patient had the first tumor in the pelvis and another one in the sphenoidal bone.

For the 2nd metachronous location of the GCT, 12 cases were in the upper limb: 7 in the humerus (4 proximal and 3 distal), 2 in the radius (1 proximal and 1 distal), 2 in the proximal ulna, and 1 in the hand. 11 cases were in the lower limb: 3 in the femur (1 proximal and 2 distal), 6 in the tibia (6 proximal and 1 distal), and 1 in the foot. In 7 metachronous tumors, the second location was in the trunk: 1 in the spine, 4 in the pelvis, and 2 in the skull. There were 7 metachronous GCTs in 2 or more locations: 1 in the proximal tibia and acetabulum, 1 in the ipsilateral proximal femur and proximal tibia, 1 in the ipsilateral proximal femur and fibula, 1 in the distal tibia and sacrum, 1 in the left proximal femur and right iliac wing, 1 in right foot, left fibula, right radius, and lung, and 1 in the pelvis, skull, and lung.

There was a 3rd metachronous location of the GCT in 11 patients (30%) and a 4th one in 2 patients (5%). 11 patients (28.5%) had a recurrence of their initial GCT at the same location. The mean duration separating the 2 locations of the metachronous multifocal tumors was 74.5 months.

The treatment used for the first location(s) of the GCT was resection of the tumor in 11 cases (29.7%), curettage with bone graft in 16 patients (43.6%), curettage alone in 1 patient (2.7%), curettage with PMMA in 2 patients (5.4%), curettage with cryotherapy in 1 patient (2.7%), amputation in 3 cases (8.1%), radiotherapy in 2 cases (5.4%), and unknown in 1 case (2.7%).

On an average 73-month followup, 20 patients (54%) suffering from multifocal metachronous GCT were disease-free, 8 patients (22%) still had a tumor in 1 or more locations but were asymptomatic, 2 patients (5%) died from the disease and its complications, and 7 patients (19%) were lost to followup.

Overall, 3 patients had metastases in the lung at the final followup: 2 as a second location and 1 as a 4th location. The 3 patients died from this complication. The mean lapse of time between the first and second locations of the GCT is 10 months.

## 5. Discussion

Giant cell tumors are typically lesions of young and middle-aged adults, with 80% of tumors occurring in patients between the ages of 20 and 50 years, and a peak prevalence in the third decade of life. They account for 4% to 5% of primary bone tumors. Multifocal GCTs are rare. Approximately 1% of cases present as multiple synchronous or metachronous lesions [17]. Most multifocal GCTs are synchronous and 68% of cases of multicentric giant cell tumor occur in less than 4 years from the initial lesion treatment [18]. They have a more aggressive course, including an increased incidence of pathologic fractures [13].

There is a slight female predominance in metachronous GCT (57% versus 43%) [3] but not a 2 : 1 ratio as reported in the literature [23]. We have found a 3 : 2 female : male ratio in this study. GCT occurs between the 3rd and 5th decades of life and >80% of patients are more than 25 years old [21, 23]. However, patients with multicentric GCT tend to be younger averaging 23 with more than 70% aged 25 years old or younger at the time of initial diagnosis. The youngest patient reported with multicentric is 10 years old [13, 16].

The etiology of multifocal GCT is unclear: de novo formation or a metastatic phenomenon. Solitary benign GCTs may metastasize to the lung or undergo malignant transformation (either de novo or following irradiation); however, pathologic analysis of multifocal GCT reveals findings identical to histologically benign solitary tumors [3]. This suggests that the multifocality of some GCT is not a metastatic phenomenon but rather represents the separate development of the tumor at multiple sites [1, 2]. Iatrogenic seeding may represent a cause of multicentric giant cell tumors [18].

The most common site of the primary GCT is around the knee (44%), followed by wrist (23%) and hand and feet (13%), and is consistent with localization of solitary GCT. Diaphyseal involvement is more found in multifocal than in solitary GCT [21]. Some studies suggested that GCTs of hand and feet are more likely to have a more aggressive course (17% in multicentric GCT compared to 2% in solitary GCT [23]). They recommended a skeletal survey for these tumors as well as multiple followups to detect metachronous GCT [24, 25].

Recurrence rate of multicentric GCT is 28.5% and is comparable to the 35% recurrence rate of solitary GCT [3, 23]. Pulmonary metastasis in solitary GCT occurs in less than 2% of patients [23]. In multicentric multifocal GCT, it occurs more frequently and averages around 8%.

In general, multicentric giant cell tumor is histologically indistinguishable from solitary giant cell tumor [21, 23] and has the following characteristics: large vascular lacunae separated by septa in which numerous giant cells are found and filled with clotted blood (blood-filled spaces with bland fibrous connective tissue septa). These cavernous spaces vessels lack walls and normal features of blood vessels and stroma is formed of histiocytes, fibroblasts, scattered giant cells, hemosiderin, and occasional inflammatory cells [18]. Differential diagnosis for multicentric giant cell tumors includes brown tumor, Paget's disease, osteomyelitis, fibrous dysplasia, giant cell reparative granuloma, Langerhans cell histiocytosis, osteosarcoma, hematopoietic malignant tumor, and metastasis [18, 23]. Before a diagnosis of multicentric giant cell tumor can be made, it is necessary to rule out the presence of hyperparathyroidism, which can produce features of a polyostotic osteolytic lesion that are virtually identical to those of a giant cell tumor of bone [4].

Limitations to this review are that only case reports are available and many patients were lost to subsequent followup to uniform the population.

## 6. Conclusion

In summary, multicentric giant cell tumor is rare and most commonly affects long bones, particularly those around the knee. It tends to occur in younger patients and frequently manifests as synchronous lesions. In addition, lesions of multicentric giant cell tumor may have an unusual metaphysodiaphyseal location. Virtually all tumors have areas with typical histopathologic features of giant cell tumor. As in solitary giant cell tumor, the most aggressive behavior of the vast majority of multicentric giant cell tumors is local recurrence, especially in multicentric metachronous GCT of hand and feet, although there have been rare cases of metastasis to the lungs. Because a variety of other primary bone lesions may also have a polyostotic presentation, the correct diagnosis relies on correlation of clinical and radiographic findings with confirmation of the diagnosis by histopathologic examination.

## Figures and Tables

**Table 1:** Analysis of all metachronous multicentric GCT.

Author	No. of cases	Age at diagnosis/sex	First location	Treatment undergone	Time between 1st and 2nd GCT	Second location	Treatment undergone	Followup	Duration
Kimball and Desanto (1958) [5]	1	39/F	Left distal ulna	Ulna resection	4 y	(1) Right distal humerus (2) Frontal bone (3) Lung metastases	Biopsy and curettage Intensive radiotherapy	Death	3 mo
Jacobs (1972) [6]	1	20/M	Proximal tibia	Radiotherapy	9 y	Right acetabulum	Curettage	N/A	N/A
Sybrandy and de la Fuente (1973) [7]	1	53/F	Left distal femur	Excision, autoclaving, and then reimplantation of the bone	2 y	Trochanteric area of the right femur	Removal of tumor + arthrodesis of hip + radiotherapy (6000 cGy)	Good general condition, walks with crutches	N/A
Tornberg et al. (1975) [8]	1	35/M	R proximal fibula	Excision + iliac bone graft	2 y	Right tibial plateau + left proximal fibula	En bloc resection	Independent + pain-free	3 y
Sim et al. (1977) [9]	9	20/M	L distal femur	Curettage + bone graft		(1) L proximal tibia/L distal tibia (2) L distal ulna (3) Recurrence in L proximal tibia	(1) Curettage/observation (2) Resection (3) Amputation	Disease-free	23 mo
		21/F	L lower cuneiform	Below knee amputation	16 y	L1 vertebra	Excision + anterior fusion	Good	5 y
		29/F	L proximal tibia	Cryotherapy + curettage + bone graft	2 y	L distal femur	Curettage + graft	Disease-free	10 mo
		24/M	L distal ulna	Curettage and then resection	2 y 5 y 11 y	(1) L prox humerus (2) C3 (3) R distal ulna	(1) Resection + hemiarthroplasty (2) Radiotherapy + fusion (3) Resection	Disease-free	25 y
		21/F	R proximal humerus	Resection with Neer prosthesis	2 y	R proximal ilium	Resection	Disease-free	8 y
		19/F	Sphenoid	Subtotal excision + radiotherapy	3 mo	(1) R proximal tibia (2) L distal radius	(1) Curettage + bone graft (2) Curettage + bone graft	Disease-free, severe neurologic sequelae	N/A
		21/F	L distal radius	Curettage + bone graft	10 mo	L proximal radius	Curettage	Disease-free	15 mo
Peimer et al. (1980) [10]	5	30/M	R ulnar head	En bloc resection	11 y 12 y	(1) R olecranon (2) Carpal + metacarpal bones	(1) Curettage + graft (2) En bloc resection	N/A	
		20/F	L proximal 4th phalanx	Curettage + graft	7 y	(1) L 3rd phalanx (2) L proximal humerus	(1) Curettage + graft (2) Curettage + graft	Recurrence in phalanx → hand amputation	N/A
		17/F	R tibia	Curettage + graft	10 mo	L hallux	Curettage	Disease-free	N/A
		18/F	L radius	Resection + graft	3 y	R 1st distal phalanx	Subtotal amputation	Disease-free	N/A
Rock et al. (1984) [11]	1	50/M	L tibia	Curettage + graft then amputation for recurrence	10 y	(1) Pelvis (2) Scalp (3) Lung	Resection Resection Radiotherapy/chemotherapy (doxorubicin + cyclophosphamide)	Death	1 y
Williams (1989) [12]	1	26/M	L distal femur	Above knee amputation (associated with osteomyelitis)	16 y	L proximal femur (pathological fracture) + R iliac wing	Resection of the proximal femur Curettage	N/A	N/A
Ogihara et al. (1994) [2]	1	29/F	L proximal humerus	Curettage + bone grafting and then en bloc resection for recurrence	20 y	Right proximal humerus	Curettage, cryotherapy, and bone graft and then en bloc resection for recurrence	Disease-free	N/A
Hindman et al. (1994) [13]	5	22/M	P1 of the L ring finger	Curettage + grafting	3 y	R calcaneum + metastatic lung disease + L fibula + R radius	Below R knee amputation + resection of the lung lesion	Recurrence in the L calcaneum	N/A
		17/F	R proximal humerus	Curettage + grafting	4 y	R distal radius	Curettage + bone graft	Recurrence in R distal radius	23 y (treatment N/A)
		10/F	L distal femur	Packing with bone graft	6 y	L proximal tibia	Resection of the proximal tibia/distal femur + prosthesis	N/A	N/A
		27/M	Distal radius	N/A	15 y	Distal humerus (same arm)	Curettage + PMMA	N/A	N/A
Bacchini et al. (1995) [14]	1	22/F	R distal femur	Curettage + autologous bone graft	2 y 7 y	(1) R distal femur, proximal femur, and proximal fibula (2) + R distal tibia	(1) Observation (2) Curettage + graft + cement (proximal tibia)/curettage + graft (distal tibia)	N/A	N/A
Cummins et al. (1996) [15]	5	16/F	R talus	Curettage + autologous bone graft	3 y	(1) R distal tibia (2) R medial tibial plateau	(1) Below knee amputation (2) Above knee amputation	Disease-free	12 y
		22/M	R fibular head	En bloc resection	2 y	R distal femur	Curettage + PMMA	Disease-free	7 y
		14/F	L proximal tibia	Curettage + autologous graft	2 y	R occipital lesion	Radiotherapy + chemotherapy	Disease-free	16 y
		18/M	L distal femur	Curettage + autologous graft	2 y 5 y	(1) R proximal tibia (2) Humeral head (R + L)	(1) Curettage + graft and then resection + knee arthrodesis for recurrence (2) Curettage + graft	Lost to followup	
Park et al. (1999) [4]	1	25/M	L distal ulna	Resection of the distal segment of the ulna	10 y	L proximal ulna	Curettage + bone chips filling	Recurrence at 2 y → total removal L ulna	N/A
Mondal et al. (2001) [16]	1	10/M	R proximal humerus	Curettage + graft	4 y	R proximal tibia	Curettage + PMMA	Disease-free	5 y
Taylor et al. (2003) [17]	1	13/M	L proximal tibia	Excisional biopsy, curettage, burring, and phenol application + PMMA	23 mo 28 mo 31 mo 40 mo 42 mo 52 mo 68 mo	(1) L distal tibia (2) L femoral head (3) L lateral femoral condyle (4) L patella (5) L distal tibia (6) L distal tibia, recurrence/fracture (7) L proximal fibula	(1) Curettage, phenol, nitrogen, and PMMA (2) Curettage, PMMA (3) Curettage, PMMA (4) Curettage, PMMA (5) Curettage, nitrogen, and PMMA (6) Resection, bone transport, and arthrodesis (7) Resection, ligamentous reconstruction	Disease-free	N/A
Haskell et al.(2003) [18]	1	23/F	R proximal tibia	Resection of proximal tibia + arthrodesis (allograft autologous graft)	24 y	L iliac wing near the sacroiliac joint	Extensive curettage + 3% hydrogen peroxide solution + reconstruction with PMMA + pins	Disease-free	3 y
Rousseau et al. (2004) [19]	1	19/F	R distal femur	Curettage + autologous bone graft	4 y 16 y 20 y 21 y	(1) R proximal tibia (2) R distal tibia + fibula (3) Recurrence in R proximal tibia + R distal fibula (4) Recurrence in R proximal tibia	(1) Curettage + autologous bone grafting (2) Curettage + PMMA (3) Curettage + PMMA (4) Curettage + PMMA	Disease-free	N/A
Stratil and Stacy (2005) [1]	1	15/M	L fibular head	Partial fibulectomy + curettage	1 y	L distal tibia Sacrum	Curettage + bone graft + PMMA Curettage, decompression + spinal fusion Chemotherapy	Disease-free	N/A
McKinney et al. (2006) [20]	1	44/F	R pelvic lesion	Curettage + autologous bone graft	15 y	Sphenoid bone	Subtotal resection of the sphenoid bone	Persistence of sphenoid + iliac lesions	3 mo
Zahid et al. (2010) [21]	1	15/F	R 4th metacarpal bone	Resection + reconstruction with fibular graft	18 mo 4 y	(1) 3rd + 5th metacarpal bones (2) R distal humerus	(1) Resection + reconstruction with fibular graft (2) Resection + arthrodesis	Disease-free	N/A
Yazdi et al. (2012) [22]	1	19/F	R distal radius	Resection	N/A	(1) L proximal + middle humerus (2) R sacral lesion (3) Nasopharynx/pterygoid	(1) Resection + prosthesis (2) Embolisation, debulking, and radiation therapy (3) Debulking, radiation therapy	Disease-free	1 y
This case	1	18/F	R distal humerus	Curettage + phenol + PMMA and then elbow resection + elbow arthroplasty for recurrence	11 y	L iliac bone	Curettage + phenol + alcohol + PMMA	Disease-free	1 y

F: female, Mo: months, L: left, N/A: not available, M: male, and PMMA: Polymethyl methacrylate.
